# The prevalence and incidence of progressive supranuclear palsy and corticobasal syndrome: a systematic review and meta-analysis

**DOI:** 10.1007/s00415-023-11791-2

**Published:** 2023-06-08

**Authors:** Shane Lyons, Dominic Trépel, Tim Lynch, Richard Walsh, Sean O’Dowd

**Affiliations:** 1 Department of Neurology, Tallaght University Hospital, Dublin, Ireland; 2 Institute of Memory and Cognition, Tallaght University Hospital, Dublin, Ireland; 3 The Dublin Neurological Institute, Mater Misericordiae University Hospital, Dublin, Ireland; 4 Trinity College Institute for Neuroscience, Trinity College Dublin, Dublin, Ireland; 5 Health Affairs, University College Dublin, Dublin, Ireland; 6 Academic Unit of Neurology, Trinity College Dublin, Dublin, Ireland

**Keywords:** Progressive supranuclear palsy, Corticobasal syndrome, Tauopathy, Epidemiology

## Abstract

**Introduction:**

Progressive supranuclear palsy (PSP) and corticobasal syndrome (CBS) are progressive neurodegenerative syndromes characterised by Parkinsonism with additional features including cognitive dysfunction, falls, and oculomotor abnormalities. Understanding the epidemiology of these conditions is critical to planning for future service provision.

**Methods:**

We conducted a systematic review of studies reporting incidence and prevalence of CBS and PSP. A search of the PubMed and EMBASE data bases was conducted from their date of inception to 13th July 2021. Meta-analysis of studies sharing similar methodologies was carried out to generate estimated pooled prevalence and incidence.

**Results:**

We found 32 studies meeting our criteria for inclusion. There were 20 studies with data on prevalence and 12 with incidence data of PSP. Prevalence of CBS was reported in eight studies while seven studies reported incidence. Reported estimates of prevalence for PSP ranged from 1.00 (0.9–1.1) to 18 (8–28) per 100,000 while prevalence rates for CBS ranged from 0.83 (0.1–3.0) to 25 (0–59). Incidence rates for PSP and CBS respectively ranged from 0.16 (0.07–0.39) to 2.6 per 100,000 person-years and 0.03 (0–0.18) to 0.8 (0.4–1.3) per 100,000 person-years. A random effects model meta-analysis of studies with similar methodologies yielded a pooled prevalence estimate for PSP of 6.92 (4.33–11.06, *I*^2^ = 89%, *τ*^2^ = 0.3907) and 3.91 (2.03–7.51, *I*^2^ = 72%, *τ*^2^ = 0.2573) per 100,000 for CBS.

**Conclusion:**

Studies of the epidemiology of PSP and CBS report highly heterogeneous findings. There is a need for further studies using rigorous phenotyping and the most recent diagnostic criteria to understand the true burden of these conditions.

**Supplementary Information:**

The online version contains supplementary material available at 10.1007/s00415-023-11791-2.

## Introduction

Progressive supranuclear palsy (PSP) and corticobasal syndrome (CBS) are progressive neurodegenerative syndromes which present with parkinsonism and a variety of additional features [1]. There is considerable clinical and neuropathological overlap between the two conditions [2, 3]. Both are associated with the proliferation of the four-repeat (4R) isoform tau, although a significant degree of pathological heterogeneity is recognised in both conditions [4, 5]. Initially described in 1964, the classic PSP phenotype (Richardson’s syndrome) is characterised by a supranuclear vertical gaze palsy, pseudobulbar palsy, and axial rigidity [6]. However, a broad range of phenotypes have since been recognised and the most recent diagnostic criteria recognise eight distinct phenotypes including a PSP-CBS overlap syndrome [7]. CBS is associated with an combination of asymmetrical cortical and extrapyramidal signs. Apraxia, myoclonus, and dystonia are commonly seen [3]. While some epidemiological studies have used the term corticobasal degeneration (CBD) we have used the term CBS throughout, as denoting the clinical syndrome. We have reserved the term CBD for the pathological diagnosis.

Epidemiological data on CBS and PSP are limited due to the relative rarity of the conditions; their protean presentation and the degree of pathological heterogeneity which they exhibit. However, an accurate understanding of the burden of disease is important for planning of medical services, provision of care, and the administration of disease modifying treatments. Previous studies have provided systematic analysis of the prevalence of PSP and CBS [8]. In this study, we aim to supplement and expand on previous studies by drawing together data on the prevalence and incidence of PSPS and CBS. Therefore, we performed a systematic review and meta-analysis to review and synthesise the available evidence with the aim of appraising the existing literature and identifying areas in need of future study.

## Methods

### Search strategy

A systematic review was conducted according to a pre-determined protocol based on the PRISMA statement for systematic review and meta-analysis (see Supplementary Table 3). A search of PubMed and EMBASE from their date of inception to 13th July 2021 was performed using the search strategies detailed below (Table 1). The search was restricted to studies in English. The study protocol was registered with the PROSPERO registry before the initial review of the titles and abstracts (crd.york.ac.uk/PROSPERO/; registration number: CRD 42021266193) [9].

**Table 1:** Details of search strategies used

EMBASE
1. progressive supranuclear palsy/exp 2. corticobasal degeneration/exp 3. corticobasal syndrome/exp 4. tauopathy/exp 5. atypical parkinsonism/exp 6. atypical parkinsonian syndrome/exp 7. 1 OR 2 OR 3 OR 4 OR 5 OR 6 8. epidemiology/exp 9. incidence/exp 10. prevalence/exp 11. 8 OR 9 OR 10 12. 7 AND 11
Pubmed
1. “progressive supranuclear palsy”[All Fields] 2. “corticobasal syndrome”[All Fields] 3. “corticobasal degeneration” [All Fields] 4. “tauopathy” [All Fields] 5. “atypical parkinsonism” [All Fields] 6. 1 OR 2 OR 3 OR 4 OR 5 7. “incidence”[All Fields] 8. “prevalence”[All Fields] 9. “epidemiology” [All Fields] 10. “population-based” [All Fields] 11. 7 OR 8 OR 9 OR 10 12. 7 AND 11

### Study selection

Studies were screened by the first author (SL). Where it was unclear whether to include a study, the senior author (SOD) acted as arbiter. No automated tools were used in the selection of studies. For the purposes of the systematic review portion of the study, we adopted broad inclusion criteria defined using the CoCoPop model (Supplementary Table 1) [10]. We considered any study which attempted to enumerate neurodegenerative disease in a general population and which reported cases of PSP or CBS as part of that enumeration. Studies were excluded if cases of PSP or CBS were not reported or could not be calculated from reported data. We considered a prevalence study to consist of a study of a general population which attempted to identify cases of PSP/CBS in a comprehensive way (for example by multiple source referrals within a defined population, screening of medical records within a region, or a standardised system of notification) and reported prevalence or reported data such that prevalence could be calculated. We considered an incidence study to consist of a study of a general population in which a comprehensive attempt was made to ascertain all cases of PSP and CBS within a defined time-period, and incidence was reported, or data reported such that incidence could be calculated. We included in the systematic review studies that identified PSP and CBS in a variety of ways: (i) in person examination of patients with identification based on published guidelines or diagnosis by expert clinicians, (ii) identification from notes and letters detailing patient diagnosis and (iii) notification to registries by treating physicians, (iv) association of patients with an appropriate code or entry in database and registry studies. Papers and conference abstracts meeting these criteria were selected for full-text review. The references of included articles and relevant review articles were hand searched for additional articles. All additional articles were evaluated in the same manner as those identified in the initial search. When studies were identified in both abstract and paper format, the paper format was included as representing a more complete data set.

### Data extraction and study quality

Data was extracted from articles using a standardised data collection form by the first authors (SL) in cases where data the relevant data was unclear or questionable, these cases were reviewed with the senior author (SOD). Studies were recorded as reporting data on prevalence and/or incidence of PSP, CBS, or both. If multiple articles reported data on the same study population the most comprehensive data were utilised. In cases where multiple studies reported prevalence or incidence data for the same geographic area at different time points all studies were included as separate estimates. Demographic data extracted included age, sex, and study location. Diagnostic criteria used, data on phenotypic subtypes, and methods of case recruitment and identification were recorded. The number of cases of PSP or CBS, and the size of the population studied, and/or person-years used to calculate incidence rates were extracted. Incidence and/or prevalence estimates from each study were extracted and reported per 100,000 population or 100,000 person-years as applicable. Study quality was assessed using the Joanna Briggs Inventory (Supplementary Table 4) [11].

### Data analysis

In cases where prevalence or incidence was not reported but could be calculated from given data this was performed. 95% confidence intervals were not reported, these were calculated assuming a binomial distribution. All statistical analyses were carried out in R (version 4.1.1 the R Foundation for statistical computing, Vienna, Austria). The *forestploter* package was employed to produce unweighted forest plots for incidence and prevalence in existing studies. Reported figures for incidence and prevalence were sub grouped by region.

Meta-analysis was limited to a subset of studies with the following characteristics: (1) full articles, excluding abstracts, (2) were designed to investigate PSP/CBS, parkinsonism, Parkinson’s disease, or FTLD. Studies of dementia were excluded as they may fail to identify movement-predominant presentations, (3) data on individual cases accessed either by review of clinical notes or examination of participants (as opposed to databases or registries) (4) did not restrict recruitment by age beyond that required by diagnostic criteria (i.e. 40 years of age) (5) specified established diagnostic criteria/guidelines, excluding the 2017 MDS Diagnostic Criteria (these were used only in one study and the expansion of the phenotype recognised by these criteria makes meta-analysis unreliable), (6) studies based on sampling were excluded.

Number of cases (numerator) and population and/or person-years (denominator) in included studies were used to calculate pooled prevalence and incidence of PSP and CBS using a random effects model. Meta-analyses were conducted using the *metafor* package [12]. Heterogeneity was assessed by the *X*^2^ test on Cochrane’s Q statistic, which was quantified by *I*^2^, assuming *I*^2^ values of 25%, 50%, and 75% respectively representing low, medium, and high heterogeneity [13]. Outliers were identified using studentised residuals. We conducted a leave-one-out sensitivity analysis to explore the sensitivity of the pooled prevalence estimates to the exclusion of individual studies.

## Results

### Identification and description of studies

**Fig.1:**
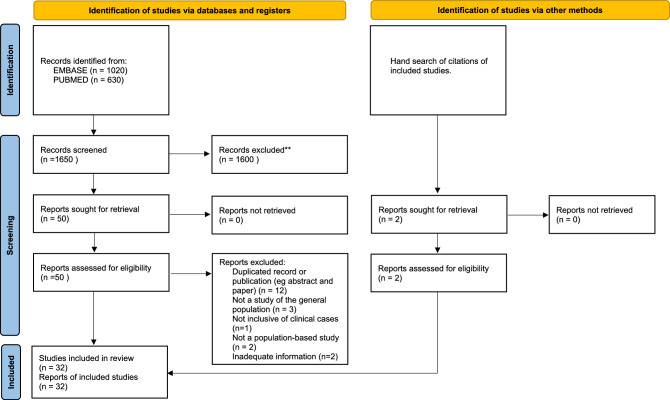
Flowchart of study identification and inclusion

The search strategy yielded 1,650 citations (1,020 from EMBASE, 630 from PubMed) (Fig. 1) carried out between 1988 and 2021. Of the studies reviewed, 32 reporting the incidence and/or prevalence of PSP and/or CBS were included in our systematic review [14–45]. Nine studies reported data on both conditions. A variety of ascertainment methods, often overlapping within a single study, were used (see Table 2 for summary details of study contents and Supplementary Table 2 and 3 for more details, Supplementary Table 6 contains a complete list of included studies). Seven studies assessed populations of fewer than 20,000 people (Supplementary Table 1 and 2).

**Table 2:** Key properties of included studies

Study		Case definition	Population definition	Cases		Condition studied
				PSP	CBS	
1	Radhnakrisnan 1988	Residents diagnosed with PSP based on clinical features of supranuclear palsy of vertical gaze, axial dystonia in extension, and pseudobulbar palsy with dysarthria and dysphagia between January 1983 and December 1986	Population of Benghazi, Libya	6		Multiple neuro conditions
2	Golbe 1988	Residents alive on 1st May 1986 who met outlined criteria for PSP as follows: all of onset after 40, progressive course, bradykinesia, supranuclear gaze palsy with 3 of: dysarthria or dysphagia, axial > limb rigidity, neck in a posture of extension, tremor minimal or absent, frequent falls or early gait disturbance, pyramidal tract signs without early or prominent cerebellar signs, unexplained polyneuropathy, dysautonomia other than isolated postural hypotension	Residents of Middlesex and Somerset County, New Jersey, USA based on population data from the 1980 US Census	11		PSP
3	De Rijk 1995	Residents alive in June 1993, who screened positive for parkinsonism and had PSP confirmed (diagnostic criteria not specified) following structured evaluation by study physicians	Residents of Ommoord, a Rotterdam suburb, aged over 55 in June 1993	1		PD
4	Bower 1997	Residents ascertained by H-ICDA codes of parkinsonism and neurodegenerative disease and diagnosed with PSP based on criteria by Collins et al. by study neurologists between 1st January 1976 and 31st December 1990	Residents of Olmsted County between 1976–1990	16		PSP and MSA
5	Wermuth 1997	Residents with parkinsonism or using dopaminergic medication and subsequently found to have Richardson’s syndrome on assessment with a study neurologist who were alive on 1st of July 1995	Residents of the Faroe Islands alive on 1st of July 1995	2		PD
6	Chio 1998	Residents identified by ICD codes of Parkinson’s disease and dopaminergic medication use found to have PSP on review with a study neurologist who were alive on 20th October 1991	Residents of the Socio-Sanitary District of Cossato alive on 20th October 1991	2		PD
7	Schrag 1999	*Prevalence*: Cases identified by screening of computer records for entries related to Parkinsonism and PSP who met NINDS-SPSP criteria on examination and review of video by study neurologists who were alive on 1st July 1997 *Incidence*: Prevalence rates divided by the mean disease duration derived from previous studies	Patients registered at 15 UK general practices in London	6		PSP and MSA
8	Nath 2001	(1) UK residents with a diagnosis of PSP notified to national surveillance services, patient advocacy, and Office of National Statistics(UK) who were alive on 1st January 1999 (2) UK residents identified by multiple screening methods, categorised as possible or probable PSP based on review of medical records and examination by study neurologist using the NINDS-SPSP criteria who were alive on 1st January 1999 (3) UK residents identified by screening of GP and hospital records with parkinsonism with PSP according to NINDS-SPSP criteria on review by study physician	(1) Population of the United Kingdom based on mid-1998 census figures (2) Regional population in the North of England based on mid-1998 census figures (3) Population served by 35 participating general practices in the North of England as reported by the Newcastle and North Tyneside Health Authority	(1) 577 (2) 80 (3) 17		PSP
9	Yamada 2001	Participants screening positive for cognitive impairment on a screening exam and subsequently diagnosed with PSP on examination by study neurologist (criteria not reported) alive on 1st January 1998	Residents of Amino-cho, Kyoto prefecture who were alive on 1st January 1998	1		Dementia
10	Harvey 2003	Residents with a diagnosis of dementia, where the illness began before the age of 65. Diagnosis of CBS based on psychiatrist review of medical records	Residents of four London boroughs aged 30–64		2	Dementia
11	Zhang 2003	Residents screened positive for parkinsonism and were subsequently found to have PSP (by criteria from Collins et al.) on review by a study physician	Residents of 27 geographically defined communities in Greater Beijing, China aged over 55, and alive on 31st December 1997	1		Parkinsonism
12	Bergareche 2004	Cases who screened positive for Parkinsonism and were subsequently identified as having PSP on review by a study physician alive on prevalence day in 1996	A random sample of 2000 people aged 65 or older in two municipalities in the Bidasao region of the Basque Country	1		Parkinsonism
13	Kawashima 2004	Participants with PSP diagnosed by neurologists or identified as having supranuclear gaze palsy or parkinsonism with dementia and subsequently diagnosed based on the NINDS-SPSP criteria by a study neurologist	Residents of Yonago City, Japan alive on 1st April 1999	8		PSP
14	Tan 2004	Participants who screened positive for Parkinson’s disease and subsequently diagnosed with PSP or CBD by a study Movement Disorders specialist using the criteria of NIND-SPSP and Kumar et al	Residents of 4 districts in Singapore aged 50 years or above	1		PD
15	Wermuth 2008	Residents identified as having Parkinsonism with a diagnosis of PSP (by NINDS-SPSP criteria) or CBS (based on criteria by Riley et al.) on review by a study neurologist who were alive on 1st July 2005	Population of the Faroe Islands based on the Faroese Registry of Residents 1st January 2005	2		PD
16	Linder 2010	Residents between 1st January 2004 and 31st December 2007 with parkinsonism and PSP (NINDS-SPSP criteria) or CBS (Litvan et al. criteria) on review by a study movement disorders specialist	Residents of the catchment of Umea University Hospital, Sweden, between 1st January 2004 and 31st December 2007	6		Parkinsonism
17	Tatari 2010	Patients diagnosed with PSP	Residents of Buenos Aires	NR		Parkinsonism
18	Winter 2010	Residents with parkinsonism and diagnosis of PSP (based on NINDS-SPSP criteria) or CBS (based on criteria by Lang et al.) following evaluation by a study physician with a first diagnosis between 1st July 2006 and 31st December 2008	Residents of the northeast district of Moscow between 1st July 2006 and 31st December 2008	5	1	Parkinsonism
19	Osaki 2011	Residents who were alive on 1st of November 2007with a diagnosis or suspicion of a Parkinsonian condition with a diagnosis of PSP (by NINDS-SPSP criteria) or CBS (by Kumar et al. criteria) on evaluation by a study neurologist	Residents of Koban district based on the census of 1st October 2005	12	6	Parkinsonism
20	Nakashita 2011	Residents > 65 with parkinsonism on door-to-door screening and a diagnosis of PSP (based on NINDS-SPSP criteria) who were alive on 1st October 2010	Residents of the town of Ama-cho, aged 65 or older, alive and legally residing in the town on 1st October 2009	1		Parkinsonism
21	Savica 2013	Residents identified in computer screening for Parkinsonism and specific Parkinsonian disorders with the diagnosis of PSP (based on criteria by Collins et al.) or CBD (based on criteria by Maraganore et al.) confirmed on review of records by a study movement disorders specialist where the initial diagnosis was made between 1st January 1991 to 31st December 2005	Residents of Olmsted County, Minnesota from 1st January 1991 to 31st December 2005	16	4	Parkinsonism
22	Caslake 2014	Participants with suspected new onset parkinsonism between April 2006 and April 2009 with a diagnosis of PSP (based on NINDS-SPSP criteria) or CBS (criteria not specified) following assessment by a study physician	People registered with one of 37 primary care practices in Aberdeen between April 2006 and April 2009	20	2	Parkinsonism
23	Withall 2014	Residents with a history of memory, behavioural, and/or language symptoms before the age of 65 years and persistent cognitive impairment for at least 6 months with confirmation of PSP/CBS diagnosis based on medical note review using DSM-IV criteria	Residents of four local government areas in Eastern Sydney < 65 years of age	3	1	Dementia
24	Khedr 2015	Participants who scored positive for parkinsonism on a screening questionnaire and had a diagnosis of PSP/CBS confirmed on review by a study neurologist according to DSM-IV criteria (CBS) and based on criteria by Boeve et al. (PSP) who were alive on 31st August 2013	A random sample of households in 10 areas of the Qena governate, Egypt		2	Parkinsonism
25	Coyle-Gilchrist 2016	*Prevalence*: Residents with a diagnosis of frontotemporal lobar degeneration including PSP (by NNIPPS criteria) or CBS (by Armstrong criteria) alive on 1st January 2014 *Incidence*: Residents with a diagnosis of frontotemporal lobar degeneration including PSP (by NNIPPS criteria) or CBS (by Armstrong criteria) first diagnosed between 1st January 2013 and 31st December 2014	The population of a catchment area including Cambridgeshire and Norfolk according to the 2013 UK Office for National Statistics midyear estimate	48	48	FTLD
26	Takigawa 2016	Residents alive on 1st October 2010 and with diagnoses of Parkinson’s disease or atypical Parkinsonism and diagnosis of PSP (based on NINDS-SPSP criteria or by Williams et al. for variant subtypes) at interview with a study neurologist	Residents of Yonago City, Japan who were alive on 1st October 2010	25		PSP
27	Fleury 2018	*Prevalence*: Residents diagnosed with parkinsonism including PSP (according to NINS-SPSP criteria) and CBS (by Armstrong et al. criteria) who were alive on 1st January 2013 (in the case of prevalence) *Incidence*: Residents who were diagnosed with parkinsonism including PSP (according to NINS-SPSP criteria) and CBS (by Armstrong et al. criteria) diagnosed between 1st January 2009 to 31st December 2012 (in the case of incidence)	Residents of the canton of Geneva from 1st January 2003 to 31st December 2012	39	14	Parkinsonism
28	Calo-Perxas 2019	Diagnoses of PSP reported to the Registry of Dementias of Girona (ReDeGi), between 2007 and 2016	Population served by the Health Region of Girona in Northeastern Catalonia between based on census figures between 2007 and 2016	33		Uncommon Dementias including PSP and CBS
29	Logroscino 2019	Residents with new diagnoses of frontotemporal lobar degeneration between 1st January 2017 and 31st December 2017, with diagnoses of PSP (defined by NINDS-SPSP criteria) or CBS (Armstrong et al. criteria) confirmed on review by a study dementia expert	Residents of Lecce and Brescia between 1st January 2017 and 31st December 2017	9	7	FTLD
30	Stang 2020	Residents with new diagnoses of Parkinsonism from 1st January 1991 to 31st December 2005 and subsequently diagnosed as PSP (by MDS 2017 criteria) or CBS (by Armstrong et al. criteria) by review of medical records by a study movement disorders expert	Residents of Olmsted County, Minnesota with the person-years at risk extrapolated from the Rochester Epidemiology Project	8	3	PSP/CBS
31	Viscidi 2020	Patients in a national electronic medical record database with 1 or more codes for PSP	Patients registered in a national electronic medical record database (CPRD GOLD)	704		PSP
32	Viscidi 2021	Patients registered in the MarketScan databases with ≥ 1 month of enrolment between 1st October 2015 and 31st October 2017 with ICD-10 (G23.1) and ICD-9 (333.0) codes corresponding to a diagnosis of PSP	People registered in large set of US insurance databases with a month or more of enrolment between 1st October 2015 and 31st October 2017	630		PSP

*CBS* Corticobasal Syndrome, *DSM-IV* Diagnostic and Statistical Manual IV, *FTLD* frontotemporal lobar degeneration, *H-ICDA* Hospital adaptation of the International Classification of Diseases, *ICD-9* International Classification of Disease, Ninth Revision, *ICD-10* International Classification of Diseases, Revision 10, *NINDS-SPSP* National Institute of Neurological Disorders and Stroke and the Society for PSP, *NJ* New Jersey, *NNIPPS* The Natural History and Neuroprotection in Parkinson Plus Syndromes, *PSP* Progressive Supranuclear Palsy, *UK* United Kingdom, *USA* United States of America

### Studies reporting the prevalence of PSP and CBS

Prevalence data on PSP and/or CBS was reported in 23 studies. This included 20 papers and three conference abstracts. 15 studies reported prevalence data on PSP only, three reported on CBS only, and five reported data on both PSP and CBS. One paper reported prevalence figures for PSP in three nested populations using a “Russian doll” design, thus, there were 22 estimates of PSP prevalence. Studies identified PSP/CBS in the context of a range of conditions as follows: PSP/CBS specifically (*n* = 7), parkinsonism (*n* = 7), Parkinson’s disease (*n* = 5), dementia (*n* = 3), frontotemporal lobar degeneration (*n* = 1).

**Fig. 2:**
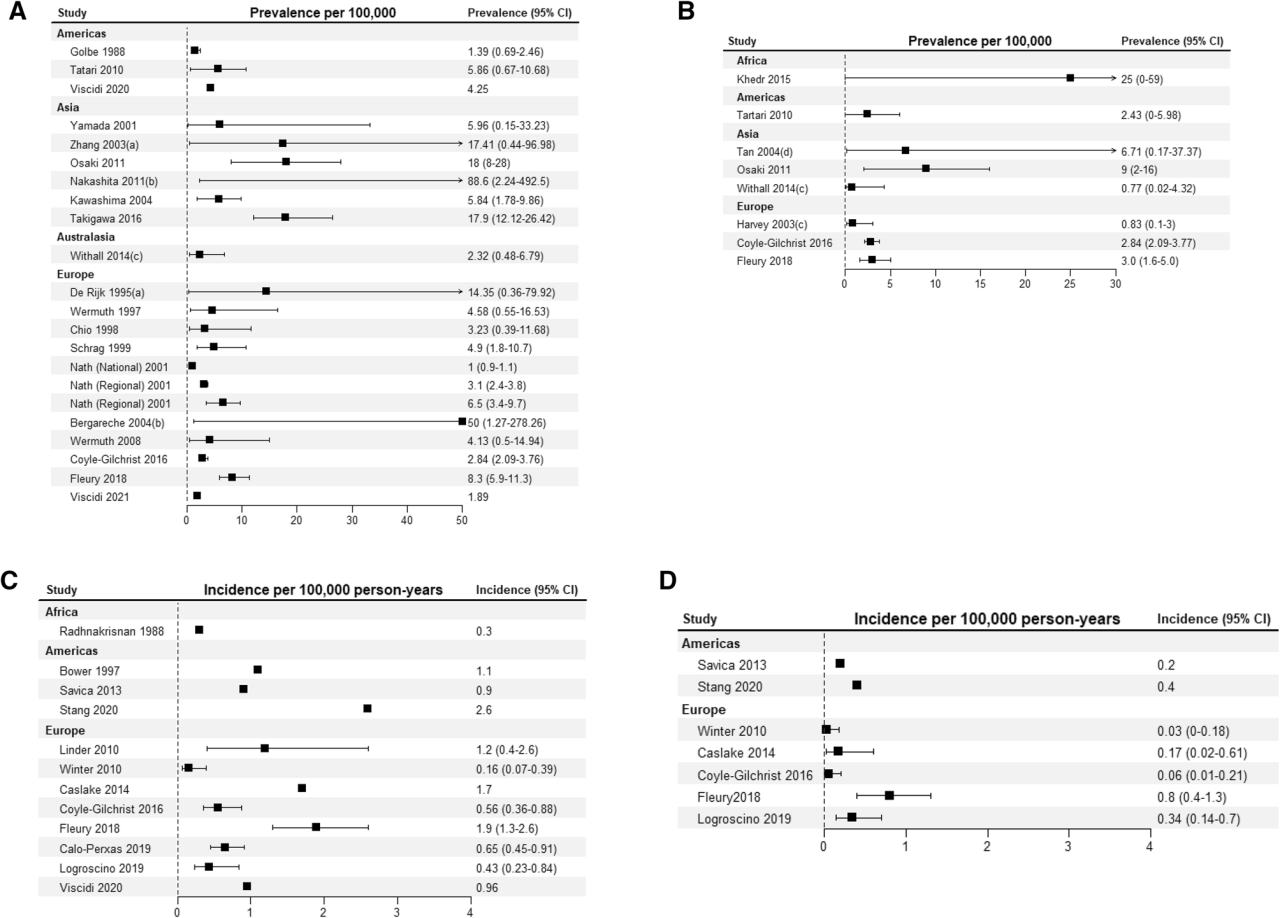
Unweighted forest plots of crude prevalence and incidence rates for: **A** PSP and **B** CBS prevalence per 100,000 population, **C** PSP and **D** CBS incidence per 100,000 patient-years with studies reported by region. **a** = Study restricted to population > 55, **b** = Study restricted to population > 65, **c** = Study restricted to population < 65, **d**  = Study restricted to population > 50. In cases where rates were not reported but could be calculated from provided data (cases and population and/or person-years) this was done

17 articles and three abstracts provided information on PSP prevalence (see Fig. 2(A) and Supplementary Table 1). Reported estimates of population prevalence ranged from 1.00 (0.9–1.1) [22] to 18 (8–28) [32] per 100,000. Age-adjusted prevalence was reported in 10 populations with estimates ranging from 1.39 to 17.26 per 100,000 [16, 39]. In a study of a large US database prevalence peaked in the age range 75–79, with a prevalence of < 2 per 100,000 before the age of 60 [45]. A UK study demonstrated a peak prevalence in the 70–74 age range [38], however, a large Swiss study reported a peak prevalence of 72.3 per 100,000 in the oldest age group (80–89 years) [40]. Prevalence data by sex was available for seven studies [16, 21, 22, 27, 32, 39, 40]. Reported prevalence figures for men ranged from 1.00 (0.9–1.1) to 25 (8–43), and from 1.0 (0.8–1.1) to 17.76 (10.38–30.39) per 100,000 for women. One study reported data on phenotypic subtypes. Richardson’s syndrome was the most common phenotype (16/25, 64%), followed by PSP-parkinsonism (3/25, 12%), and PSP-progressive gait freezing (2/25, 8%) [14]. Two studies of PSP prevalence in Yonago City, Japan conducted 11 years apart demonstrated an increase in the recorded prevalence of PSP in this area during that time (from 5.82 to 17.9 per 100,000) [27, 39]. Meta-analysis of nine studies yielded a pooled prevalence estimate of 6.92 (4.33–11.06) per 100,000, with a high degree of heterogeneity (*I*^2^ = 89%, *τ*^2^ = 0.3907, *P* < 0.01) (Fig. 4(A)). One study [38] was a significant outlier (studentised residual =  – 2.00), exclusion of this study resulted in a prevalence estimate of 8.29 (5.50–12.49). Pooled prevalence figures of relevant subgroups are available in Fig. 5(A). Pooled prevalence for three studies from Japan yielded a prevalence of 12.85 (10.97–19.68) (*I*^2^ = 76%, *τ*^2^ = 0.2969, *P* = 0.02) while six studies carried out in Europe yielded a pooled prevalence of 4.95 (3.19–7.68) (*I*^2^ = 81%, *τ*^2^ = 0.1789, *P* < 0.01).

Eight studies (seven papers and one abstract) reported the prevalence of CBS (Table 2 and Supplementary Table 2). Reported prevalence figures ranged from 0.77 (0.02–4.32) to 25 (0–59) per 100,000. Age adjusted prevalence was available for two studies of CBS with rates of 3.2 (1.5–4.8) and 6 (0–12) per 100,000 [32, 40]. Prevalence by sex were available for two studies, with male vs female prevalence figures of 2.6 vs 3.3[40], 16 vs 3 per 100,000 [32]. Meta-analysis of three studies of the prevalence of CBS yielded a pooled prevalence 3.91 (2.03- 7.51, *I*^2^ = 72%, *τ*^2^ = 0.2573, *P* = 0.03).

### Studies reporting the incidence of PSP and CBS

The incidence of PSP/CBS was reported in 12 studies (PSP only *n* = 5, PSP and CBS *n* = 7, no studies with CBS alone), including 11 papers and one abstract. Two additional studies reported an indirectly calculated incidence rate, using prevalence and survival data and were not included in the analysis of incidence [16, 21]. Included studies reported on the incidence of parkinsonism (*n* = 6), PSP and CBS specifically (*n* = 2), dementia (*n* = 1), FTLD (*n* = 2), and a single study reported on a number of uncommon neurological diseases. Age-adjusted rates were available for four studies of PSP [14, 31, 40, 45] and two for CBS [14, 40]. Three of these studies used direct standardisation to a national population with details of standardisation not reported in one study.

**Fig. 3:**
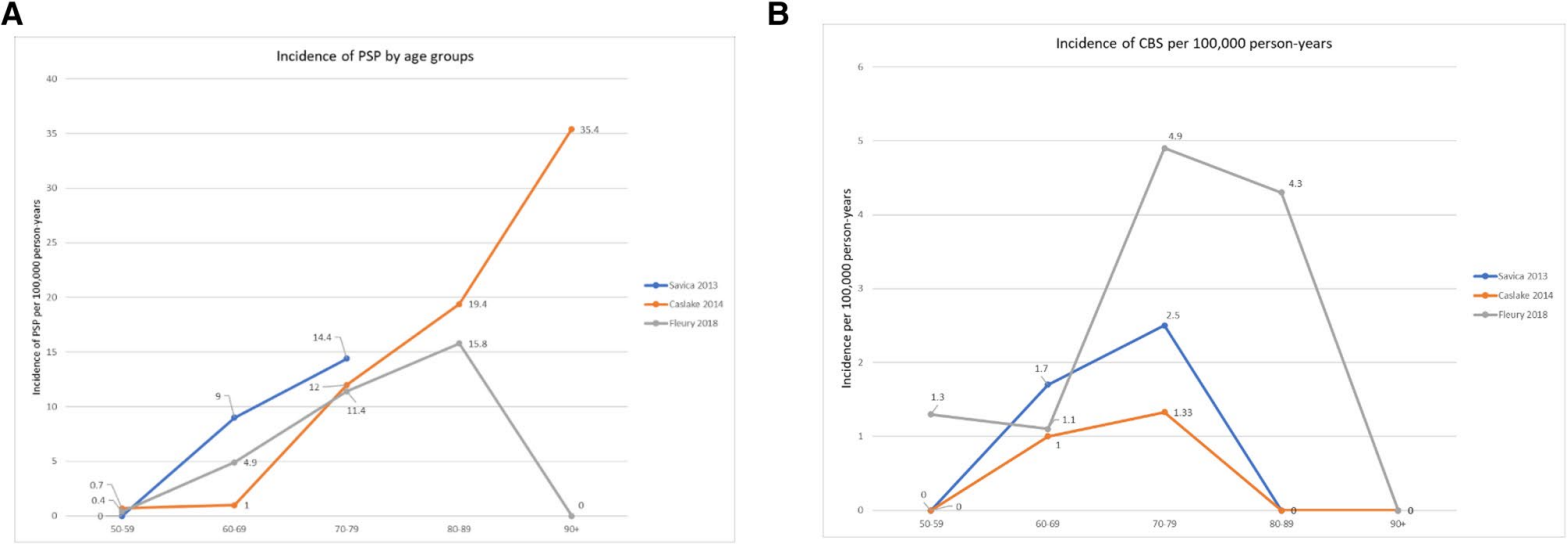
Incidence of PSP **A** and CBS **B** per 100,000 person-years by age bands in Savica (2013), Caslake (2014), and Fleury (2018)

**Fig. 4:**
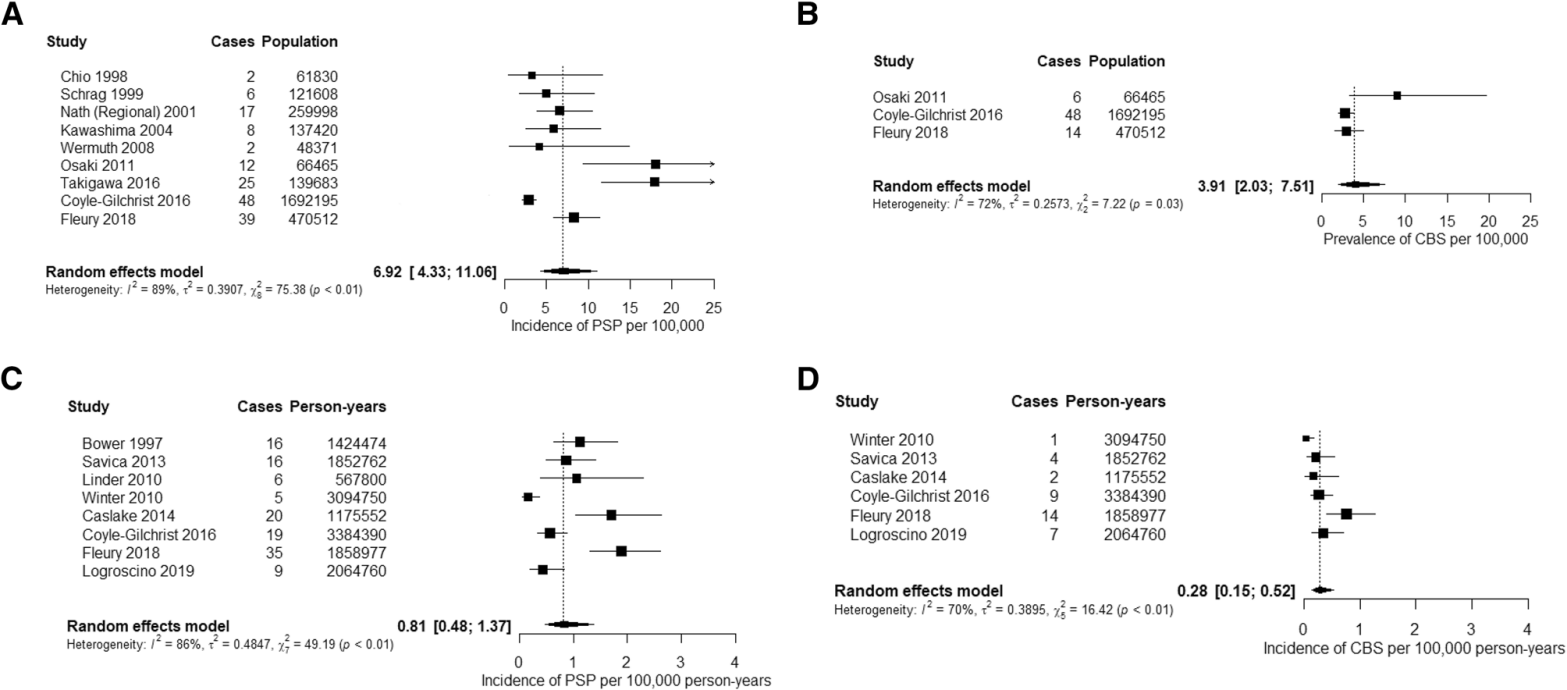
Meta-analyses of studies reporting prevalence and incidence of PSP and/or CBS

Reported incidence rates for PSP ranged from 0.3 to 2.6 per 100,000 person-years (see Fig. 2(C) and Supplementary Table 2), while age-adjusted rates ranged from 0.12 to 2.0 per 100,000 person-years. Incidence rates in populations > 60 years ranged from 0.84 (0.27–1.96) [14] to 9.7 per 100,000 person [18] years reported. Incidence of PSP was reported in comparable age increments in three studies (Fig. 3). PSP incidence was reported by sex in eight studies. Incidence among men ranged from 0.17–3.9 per 100,000 person-years and from 0.1–0.8 per 100,000 person-years among women. Incidence of PSP was reported in studies of the population in Olmsted County, Minnesota over two time periods, the first study reported on the period 1976–1990 produced an incidence of 0.9 per 100,000 person-years [18], while a study of the 1991–2005 population using the 2017 MDS criteria returned an incidence rate of 2.6 per 100,000 person-years [43]. Meta-analysis of eight studies yielded a pooled incidence of 0.81 per 100,000 person-years (0.48–1.37, I2 = 86%, τ2 = 0.4847, p < 0.01, Fig. 4(C)). One study [14] was a significant outlier (studentised residual =  – 2.57); when this was excluded from the analysis, a pooled incidence of 0.99 (0.65–1.51) was found.

**Fig. 5:**
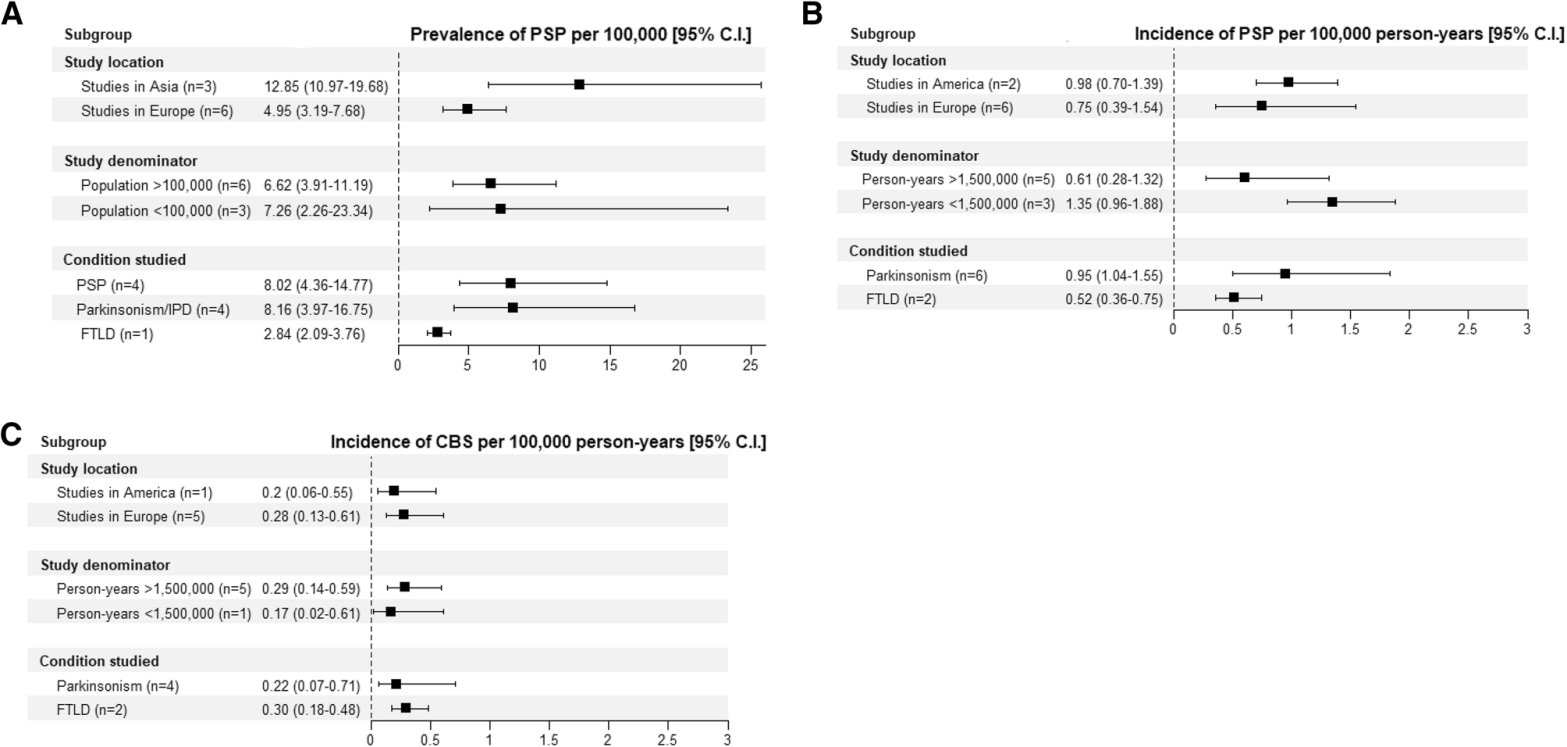
Pooled proportions in study subgroups be location, study denominator, and condition studied in **A** PSP prevalence, **B** PSP Incidence, **C** CBS incidence

The incidence of CBS ranged from 0.03 [14] to 0.8 per 100,000 person-years [18] (Fig. 2(D) and Supplementary Table 2). Age-adjusted rates were available for two studies of CBS with rates of 0.02 (0.01–0.12) [14] and 1.4 (0.3–2.4) [40] per 100,000 person-years reported. Meta-analysis of six studies yielded an incidence of 0.28 per 100,000 person-years (0.15–0.52, *I*^2^ = 70%, *τ*^2^ = 0.3896, *p* < 0.01, Fig. 4(D)). Pooled incidence figures for relevant subgroups are reported in Fig. 5(B) and (C).

## Discussion

A true and reliable description of the epidemiology of PSP and CBS remains elusive. This study reviews and synthesises existing data on the prevalence and incidence of these disorders, but significant challenges remain. Estimates of prevalence and incidence confirm that PSP and CBS are uncommon conditions but there is a high degree of heterogeneity in the existing literature. Studies generally accord with the reported preponderance of male cases in PSP, although one high quality study demonstrated an equal prevalence of male and female cases. PSP is more common in older age bands, where these are reported. Only one population-based study of PSP prevalence reported phenotypic subtypes, suggesting a predominance of Richardson’s Syndrome (64%).

There are fewer studies of the epidemiology of CBS than PSP. Most studies support the view that CBS is significantly rarer than PSP [10] although it is worth noting that in one well-designed study of the prevalence of CBS was the same as that of PSP.[38] Since the conclusion of our study selection in July 2021 several studies have added to the field. A study of a large health insurance database in Israel has provided registry data on a large population. The epidemiological advantages of this database include the fact that enrolment in a healthcare database is mandatory in Israel, it provides stable database population, and this single insurance database includes 25% of the national population [46]. Estimates from this study accord reasonably well with the most widely cited data with prevalence estimates of 5.3 per 100,000 and incidence of 1 per 100,000 for PSP as well as our pooled estimate of European studies.

### Study design and methodology

Registry and database-based studies have generally reported lower prevalence and incidence numbers than studies which have sought referral from a comprehensive array of sources [44, 45]. The 2001 study by Nath et al. demonstrates the limitations of a database and registry-based approach, with a Russian-doll model demonstrating higher prevalence figures with more granular and comprehensive methods of ascertainment [22]. While database and registry studies provide the sample size necessary to study rare diseases, they may lack the ability to accurately capture cases in disease where reliable biomarkers are lacking, such as PSP and CBS. However, studies with more complete ascertainment may be limited in size, identifying relatively few cases in smaller populations and generating estimates of incidence and prevalence with very broad confidence intervals.

### Changes in prevalence and incidence of PSP and CBS over time

The degree of heterogeneity between studies makes inferences regarding longitudinal trajectories of prevalence difficult, however, two studies conducting in Yonago City, Japan 11 years apart, demonstrated an increase in prevalence over that time, from 5.82 (1.78–9.86) to 17.9 (12.12–26.42) per 100,000 [27, 39]. This increase remained statistically significant following adjustment for age and sex. However, although PSP-RS was numerically increased this did not reach statistical significance, suggesting that increased recognition of non-RS subtypes might have played a role in this apparent trend. This may be driven by several factors which are likely to be replicated in other settings, such as an aging population and increased recognition of PSP subtypes, and some which are unique to the local context, such as the inclusion of PSP in the Japanese Specified Treatment Research Program in 2003. However, the later of these studies is a relative outlier in terms of reported prevalence and, as geographical clusters of PSP are known to occur, may not be generalisable. Studies of the population of Olmsted County, Minnesota demonstrated an incidence rate which was essentially unchanged in the periods 1976–1990 and 1991–2005. Two studies published since the conclusion of our data collection two studies have added more information on secular trends in the prevalence and incidence of PSP and CBS. Swallow et al. have demonstrated stable incidences of PSP and CBS over a 20 year period in a Northern European population [47]. Logroscino et al., whose earlier study is included in our analysis have published updated data on the incidence of a range of fronto-temporal lobar degeneration syndromes in nine countries with combined estimated incidence of PSP and CBS of 0.51 (0.22–1.20) per 100,000 person-years [48].

### Impact of diagnostic criteria on studies of PSP/CBS

Evolution in diagnostic criteria may explain a significant proportion of apparent change over time. Many existing studies used the NINDS-SPSP criteria for PSP which, despite excellent specificity, has poor sensitivity for Richardson’s syndrome early in the disease course and does not account well for variant presentations, which may be the initial presentation in up to 60–75% of PSP cases [49, 50]. Therefore, these studies may underestimate the true prevalence and incidence of PSP. In one study, only 25% of cases in which PSP was demonstrated pathologically received a PSP diagnosis at initial evaluation [50]. Two studies conducted in Olmsted County, Minnesota provide insight into the impact of the 2017 MDS criteria on PSP incidence: in a 2013 study using the NINDS-SPSP criteria the incidence of PSP was 0.9, in a 2020 study of the same population using the 2017 criteria, an incidence rate of 2.6 was reported [34, 43]. The most common criteria used in the diagnosis of CBS were the 2013 Armstrong criteria which despite usefully expanding the range of phenotypes seen in CBS may have limited specificity [51].

### Regional and socio-economic difference

In common with many other neurological conditions the epidemiology of PSP/CBS is much more extensively studied in high-income countries with Europe, America, and Japan being particular areas of activity with low-and-middle-income-countries (LMICs) underrepresented. As the prevalence of neurodegenerative disease increases with age, and the populations of many LMICs are aging[52], there is an urgent need to expand the study of PSP/CBS to these areas. In addition, of particular interest is the relatively high prevalence of PSP reported in studies from Japan, and further studies from that region may provide clues to genetic and environmental risk factors [32, 39].

### Study strengths and limitations

This study comprehensively collates and analyses the existing literature on the incidence and prevalence of PSP/CBS. A recent systematic review and meta-analysis of the prevalence of PSP and CBS by Swallow et al. identified similar issues with heterogeneity of study design and a wide range of reported prevalence figures [8]. Our study complements this work, including a discussion on data from existing registry based studies and data and meta-analysis on incidence of PSP and CBS. Our study has several limitations. There is a high degree of heterogeneity between the identified studies, which limits the ability to generalise from recorded observations, especially when the possibility of geographic or genetic clustering may occur. Many of the studies are small and underpowered to estimate the prevalence of rare conditions such as PSP and CBS. Nevertheless, significant heterogeneity remains even when such studies are excluded. Several studies restricted their recruitment by age [17, 26, 33] and these studies reported prevalence of PSP/CBS higher than unrestricted studies, consistent with an increasing prevalence of neurodegenerative disease in older age. The relatively small number of studies makes identifying trends problematic. All studies of PSP and CBS are limited by the degree of phenotypic heterogeneity and the absence of reliable antemortem biomarkers of the underlying neuropathology, and any inference derived from meta-analysis will be susceptible to similar limitations. PSP has also demonstrated a tendency to cluster for environmental reasons [53, 54] and genetic reasons [55] which may further confound attempts to generalise from existing studies, especially from small sample sizes. Lastly, because of the recognition of the wider phenotype associated with PSP and CBD pathology it is difficult to extrapolate epidemiological estimates from studies which use older diagnostic criteria.

### Future directions in the epidemiology of PSP/CBS

Future epidemiological studies of PSP and CBS face several challenges. Studies of PSP/CBS require relatively large populations to generate accurate estimates. Accurate detection of variant presentations of PSP and CBS will require collaboration of multiple specialties, including psychiatry of older age, neurology, medicine for the elderly, and family physicians. Given the possibility of evolution of clinical syndromes and consequent diagnostic instability studies should include a range of neurodegenerative diagnoses and longitudinal follow-up. Large, prospective registries of neurodegenerative diseases with comprehensive reporting requirements and including detailed clinical information might offer the best hope for accurate population level data, although the logistic and data management challenges would be formidable. In the future, with the development of fluid and imaging based biomarkers, epidemiology informed by in-vivo molecular profiles may improve the reliability of epidemiological data [56].

## Conclusion

Data regarding the prevalence and incidence of PSP and CBS are rare, with information on CBS particularly limited. Existing estimates are varied, influenced by study design and size. Evolving diagnostic criteria constitute a particular challenge in the study of these conditions. There is a need for further epidemiological studies, adequately powered to assess the epidemiology of rare diseases, with robust methods of clinical assessment using the most recent diagnostic criteria.

## Supplementary Information

Below is the link to the electronic supplementary material.

Supplementary file1 (DOCX 99 KB)
