# Supplementary material for: The prevalence and incidence of progressive supranuclear palsy and corticobasal syndrome: a systematic review and meta-analysis
Source: J Neurol. 2023 Jun 8;270(9):4451–65. doi: 10.1007/s00415-023-11791-2 (PMC10421779; doi:10.1007/s00415-023-11791-2)
Supplement: Supplementary file 1 — Supplementary file1 (DOCX 99 KB) [file 415_2023_11791_MOESM1_ESM.docx]

| **Supplementary Table 1. Inclusion criteria for systematic review using CoCoPop Model** | | |
| --- | --- | --- |
| **CoCoPop Element** | **Inclusion** | **Exclusion** |
| **Co**ndition | Studies of PSP and CBS defined in the following ways:   1. in person examination of patients with identification based on published guidelines or diagnosis by expert clinicians 2. identification from notes and letters detailing patient diagnosis 3. notification to registries by treating physicians 4. association of patients with an appropriate code or entry | Studies in which cases of PSP or CBS were not reported or could not be calculated from reported data. |
| **Co**ntext | Epidemiological studies which enumerate neurodegenerative disease which were reported in published, peer-reviewed articles or conference abstracts |  |
| **Pop**ulation | We included studies of adult populations including studies of age restricted subpopulations. | Studies of cohorts with specific environmental or genetic risk factors for neurodegenerative disease. |
| Munn Z, Stern C, Aromataris E, Lockwood, C., & Jordan, Z. (2018). What kind of systematic review should I conduct? A proposed typology and guidance for systematic reviewers in the medical and health sciences. BMC Medical Research Methodology, 18, 5. doi: 10.1186/s12874-017-0468-4. | | |

| **Supplementary Table 2. Further details of studies presenting data on the incidence and/or prevalence of PSP.** | | | | | | | | | | | | |
| --- | --- | --- | --- | --- | --- | --- | --- | --- | --- | --- | --- | --- |
|  | **Location** | **Methods to identify possible cases** | **Methods to identify included cases** | **Diagnostic Criteria** | **Years of data collection** | **Population** | **Cases** | **Incidence of PSP**  **per 100,000 (95% CI)** | | **Prevalence of PSP per 100,000 (95% CI)** | | **Subgroups reported** |
|  |  |  |  |  |  |  |  | **Crude** | **Age**  **adjusted** | **Crude** | **Age adjusted** |  |
| 1. Radhnakrisnan,  1988 | Benghazi,  Libya | Medical records | Records from clinics | Not  specified | 1983-  1986 | 519,000 | 6 | 0.3 |  |  |  |  |
|  |  | -regional polyclinics |  |  |  |  |  |  |  |  |  |  |
|  |  | -university hospitals |  |  |  |  |  |  |  |  |  |  |
|  |  | -rehabilitation centres |  |  |  |  |  |  |  |  |  |  |
|  |  | -regional neurology |  |  |  |  |  |  |  |  |  |  |
|  |  | centre |  |  |  |  |  |  |  |  |  |  |
| 2. Golbe, 1988 | New Jersey, USA | Letters sent to all neurologists in the region  Local PD support groups  Inquiries to nursing homes | Examination by study authors | Study criteria | 1986 | 799,022 | 11 |  |  | 1.38  (0.69-2.46)* | 1.39  Male: 1.53 Female: 1.23 | Male/Female  >55 |
| 3. De Rijk, 1995 | Rotterdam,  Netherlands | Invitations sent to all  residents ≥55 with  screening examination  for parkinsonism | Examination by  a neurologist of neurologist-in-training | Not  specified | 1990-  1993 | 6,969 | 1 |  |  | 14.35  (0.36-79.92)* |  | Participants >55  only |
|  |  |  |  |  |  |  |  |  |  |  |  |  |
|  |  |  |  |  |  |  |  |  |  |  |  |  |
|  |  |  |  |  |  |  |  |  |  |  |  |  |
|  |  |  |  |  |  |  |  |  |  |  |  |  |
|  |  |  |  |  |  |  |  |  |  |  |  |  |
|  |  |  |  |  |  |  |  |  |  |  |  |  |
| 4. Bower, 1997 | Olmsted  Co.,  Minnesota,  USA | Medical records  -Mayo clinic record  linkage | Review of record parkinsonism by a neurologist with movement disorders expertise  Adjudication panel of 3 neurologists for difficult cases | Collins  1995 | 1976-  1990 | 1,424,474 | 16 | 1.1  Male: 1.3  Female: 0.9 | >50: 5.3  >50 Male:6.9  >50 Female:4.1 |  |  | Male/Female  0-49  50-59  60-79  80-99  Male 0-49  Male 50-59  Male 60-79  Male 80-99  Female 0-49  Female 50-59  Female 60-79  Female 80-90 |
| 5. Wermuth, 1997 | Faroe Islands, Denmark | 1. Patients as receiving levodopa containing drugs and dopamine agonists by pharmacy records 2. Patients identified by   -National Hospital records  -GPs  -Nursing homes | Interview and clinical examination by a neurologist or neurology resident | NR | 1995 | 43,709 | 2 |  |  | 4.58  (0.55-16.53)* |  |  |
| 6. Chio, 1998 | Cossate, Italy | Medical records  -Neurologists  -GPs  Hospital admission records  Inquiries to pharmacists about PD drugs | Interview and examination by neurologist. | NR | 1991 | 61,830 | 2 |  |  | 3.23  (0.39-11.68)* |  |  |
| 7. Schrag, 1999 | London, UK | Medical records  -Screening of computerised records via NHNN GP linkage scheme | Review of records  Questionnaire and video recording with subsequent adjudication | NINDS- SPSP | 1997 | 121,608 | 6 |  |  | 4.90  (1.8-10.7)  Male: 5  Female: 4.8 | 6.4  (2.3-10.6) | Age-adjusted Male/Female |
| 8. Nath, 2001 | 1. UK 2. North of England   Newcastle- upon-Tyne, UK | (1) British Neurological Surveillance Unit PSP (Europe) Association Office of National Statistics  Referrals invited from neurologists  (2) Referrals  -neurologists  -care of the elderly physicians  -psychogeriatricians  -physicians  Correspondence review from the above group  Database screening Hospital admission data  Records of academic neurology meetings  (3) Reviewed practice records of 35 GP practices | (1)Data from medical records  (2)Review of patient records and examination where possible.  (3) Structured interview, clinical examination, and videotape | NINDS- SPSP | 1998-  2000 | (1)  59,236,500  (2)  2,589,240  (3)  259,998 | (1) 577  (2) 80  (3) 17 |  |  | (1) 1.00  (0.9-1.1)  Male: 1.00  (0.9-1.1)  Female: 1  (0.8-1.1)  (2) 3.10  (2.4-3.8)  Male: 2.4  (1.6-3.3)  Female: 3.7  (2.7-4.8)  (3) 6.5  (3.4-9.7)  Male: 6.2  (1.9-10.5)  Female: 6.9  (2.4-11.4) | 1. NA 2. 2.4   (1.9-3.0)   1. 5.0   (2.5-7.5) | Male/Female Age-adjusted |
| 9. Yamada, 2001 | Kyoto, Japan | Door-to-door screening examination of all people >65 | Brief screening exam for cognitive impairment  Neurologist examination for those who screened positive | NR | 1998 | 16,765 | 1 |  |  | 5.96  (0.15-33.23)* |  | Participants >65 |
| 10. Zhang, 2003 | Greater Beijing, China | Door-to-door screening examination of all residents >55 (96% of eligible population undergoing assessment) | Neurologist administered questionnaire and examination  Cases adjudicated by PI | Collins 1995 | 1997-  2001 | 5,743 | 1 |  |  | 17.41  (0.44-96.98)* |  | Participants >55 |
| 11.Bergareche, 2004 | Basque region, Spain | Door-to-door survey | Screening using SNES tool  Clinical assessment if screen positive | NR | 1996-  1999 | 2,000 | 1 |  |  | 50  (1.27-278.26)* |  | Patients >65 only |
| 12. Kawashima,  2004 | Yonago City, Japan | Medical records  -Hospital  Inquiries to hospital- based neurologists Study visits to nursing homes | Review by study neurologist | NINDS- SPSP | 1999-  2002 | 137,420 | 8 |  |  | 5.82  (1.78-9.86)  Male: 9.14  (1.82-16.47)  Female: 2.75  (1.08-6.65) | 5.03  Male: 7.92 Female: 2.27 | Male/Female Age-adjusted |
| 13. Wermuth,  2008 | Faroe Islands, Denmark | Patients as receiving levodopa containing drugs and dopamine agonists by pharmacy records  Invitation to patients diagnosed with PD or parkinsonism by the local neurologists at the National hospital or two regional hospitals | Examination by study neurologist or neurologist-in-training | NINDS- SPSP | 2004-  2005 | 48,371 | 2 |  |  | 4.13  (0.5-14.94)* |  |  |
| 14. Tartari, 2010 | Buenos Aires, Argentina | Medical records  -surveillance of electronic medical records within a HMO  -pharmacy system for patients prescribed antiparkinsonian  drugs | No specified | NR | NR | 148,301 | NR |  |  | 5.68  (0.67-10.68) |  |  |
| 15. Linder, 2010 | Umea, Sweden | Referrals  -general practitioners  -private practitioners  -psychiatrists  -neurosurgeons  -company health services  -nursing homes Medical records  -referral letters to neurology department screened  -nursing home records reviewed  Visit of largest nursing home institution | Examination by a study neurologist specialising in movement disorders  Independent assessment of videotape of UPDRS-III exam | NINDS- SPSP | 2004-  2007 | 141,950 | 6 | 1.1 (0.4-2.4) | 1.2 (0.4-2.6) |  |  |  |
| 16 Winter, 2010 | Moscow, Russia | Referrals  -primary care centres  -hospital personnel Medical records  -University Movement Disorders centre  -primary care centres  -hospitals | Exam performed by neurologist with 5 years experience in movement disorders | NINDS- SPSP | 2006-  2008 | 1,237,900 | 5 | 0.16  (0.07-0.39)* | 0.14  (0.08-0.21)  Male: 0.17  (0.04-0.60)  Female: 0.1  (0.01-0.45) |  |  | Age-adjusted Male/Female  0-59  60-64  65-69  ≥70  Male 0-59  Male 60-64  Male 65-69 Male ≥70 Female 0-59  Female 60-64  Female 65-69  Female ≥70 |
| 17. Osaki, 2011 | Koban district, Japan | Referrals from  -Japanese long term care insurance system  -local medical institutions | Review of records  Diagnostic visit by study physician | NINDS- SPSP | 2007 | 66,465 | 12 |  |  | 18 (8-22)  Male:25  (8-43)  Female: 12  (0-23) | 10 (2-17)  Male:14 (1-26)  Female: 6 (2-14) | Male/Female 0-39  40-49  50-59  60-69  70-79  80-89  90+  Male 0-39  Male 40-49  Male 50-59  Male 60-69  Male 70-79  Male 80-89  Male 90+  Female 0-39  Female 40-49  Female 50-59  Female 60-69  Female 70-79  Female 80-89  Female 90+ |
| 18. Nakashita,  2011 | Ama-Cho, Japan | Neurological examination of all residents ≥60 | NR | NR | 2011 | 1,129 | 1 |  |  | >60: 88.6* |  | Patients >60 only |
| 19. Savica, 2013 | Olmsted Co.,  Minnesota, USA | Medical record linkage system (Rochester Epidemiology Project) | Review of medical records by movement disorders specialist | NINDS- SPSP | 1991-  2005 | Not stated  (1,852,762 person-years)  Details of REP reported by St Sauver et al:  502,820^1^ | 16 | 0.9  Male: 1  Female: 0.6 |  |  |  |  |
| 20. Caslake, 2014 | Aberdeen, Scotland, UK | Referrals  -GPs  -neurologists  -medicine for the elderly physicians  -old-age psychiatrists  -general physicians Medical records  -screening of referral letters  -GP databases  -Hospital discharge data  Questionnaire-based population screening | Review by a neurologist with an interest in PD or a supervised trainee neurologist | NINDS- SPSP | 2002-  2006 | 317,357  (1,176,552  person- years) | 20 | 1.7 (1.0-2.4)  >65: 12.2 |  |  |  | Male/Female 0-39  40-49  50-59  60-69  70-79  80-89  90+  Male 0-39  Male 40-49  Male 50-59  Male 60-69  Male 70-79  Male 80-89  Male 90+  Female 0-39  Female 40-49  Female 50-59  Female 60-69  Female 70-79  Female 80-89  Female 90+ |
| 21. Withall 2014 | Eastern Sydney, Australia | Structured questionnaire distributed to health professionals in the region  Search of hospital records | Review of medical records | NR | 2008 | 129,070 | 3 | 2.32  (0.48-6.79)* |  |  |  | Participants <65 only |
| 22. Coyle-  Gilchrist, 2016 | Cambridge, UK | Referrals from  -Regional specialist clinics  -Clinical research networks  -Self-referral  -Local and national charities  Search of clinic databases | Examination by neurologist Structured assessments of speech, language, and cognition MRI of brain | NNIPPS | 2013-  2014 | 1,692,195 | 48 | 0.56  (0.36-0.88)* |  | 2.84  (2.09-3.76)* |  | 0-44  45-49  50-54  55-59  60-64  65-69  70-74  75-79  80-84  85-89  90+ |
| 23. Takigawa,  2016 | Yonago City, Japan | Medical records  -University hospital | Examination by a board qualified neurologist | NINDS- SPSP | 2009-  2014 | 148,271 | 25 |  |  | 17.90  (12.12-26.42)  Male: 18.05 Female: 17.76 | 17.26  Male:  18.14  Female:  16.63 | Age-adjustedMale/Female |
| 24. Fleury, 2018 | Geneva, Switzerland | Medical records  -Hospitals  -Private Neurologists  -Nursing homes | Review of clinical notes and imaging by a movement disorders specialist | NINDS- SPSP | 2003-  2012 | 470,512 | 39 | 1.9 (1.3-2.6)  Male:2.4 (1.5-3.7)  Female: 1.4  (0.7-2.3) | 2.0 (0.7-3.4)  Male: 3.9  (1.0-6.7)  Female: 0.8  (0.0-1.8) | 8.3 (5.9-11.3)  Male: 9.7  (6.1-14.6) Female: 7  (4.1-11.2) | 5.7 (3.8-7.6)  Male: 7.5  (4.3-10.6) Female: 4.6  (2.2-6.9) | Male/Female 0-39  40-49  50-59  60-69  70-79  80-89  90+  Male 0-39  Male 40-49  Male 50-59  Male 60-69  Male 70-79  Male 80-89  Male 90+  Female 0-39  Female 40-49  Female 50-59  Female 60-69  Female 70-79  Female 80-89  Female 90+ |
| 25. Caló-Perxas,  2019 | Girona, Italy | Clinical dementia registry (ReDeGi) | Diagnosis based on review by specialist physicians in participating hospitals | NR | 2007-  2016 | 753,576 | 33 | Male: 0.8  (0.5-1.2)  Female: 0.5  (0.3-0.9) | Male ≥65: 3.7  (2.2-5.8)  Female ≥65:2.2 (1.2-3.8) |  |  | Male/Female Male ≥30 to  <65  Male ≥65 Female ≥30 to  <65  Female ≥65 |
| 26. Logroscino,  2019 | Salento and Brescia, Italy | Referrals  -general practitioners  -regional network of dementia services, the Centres for the Cognitive Disorders and Dementias (CDCD) | Standardised evaluation by research team including neuropsychology, MRI, and, in selected, CSF analysis | NINDS- SPSP | 2017 | 2,070,760 | 9 | 0.43  (0.23-0.84)* |  |  |  |  |
| 27. Stang, 2020 | Olmsted Co.,  Minnesota, USA | Medical record linkage system (Rochester Epidemiology Project) | Review of clinical records by movement disorders specialist | Höglinger 2017 | 1991-  2005 | Not stated  Details of REP reported by St Sauver et al:  502,820^1^ | 8 | 2.6 | >50: 5.0  Male >50: 7.6  Female >50:3.0 |  |  | Male/Female  >50  Male >50  Female >50 |
| 28. Viscidi, 2020 | UK | Screening of national  electronic database (CPRD GOLD) | Database entries | Database codes | 1987-  2018 | 1,300,000 | 704 | 0.96 | 0.97 | 4.25 | 4.35 | Age-adjusted |
| 29. Viscidi, 2021 | USA | Medical records  -Large insurance database: IBM MarketScan  Commercial and Medicare Supplemental Databases | Database entries | ICD codes  ≥40 | 2015-  2017 | 38,053,188 | 630 |  |  | 1.89 | 2.95 | 45-49  50-54  55-59  60-64  65-69  70-74  75-79  80-84  85+ |
| Abbreviations:  CBS=Corticobasal Syndrome, DSM-IV=Diagnostic and Statistical Manual IV, FTLD=frontotemporal lobar degeneration, H-ICDA=Hospital adaptation of the International Classification of Diseases, HD=Huntington’s disease, ICD-9= International Classification of Disease, Ninth Revision, ICD-10=International Classification of Diseases, Revision 10, NINDS-SPSP=National Institute of Neurological Disorders and Stroke and the Society for PSP, NJ=New Jersey, NNIPPS=The Natural History and Neuroprotection in Parkinson Plus Syndromes, NR=Not reported, PSP=Progressive Supranuclear Palsy, UK=United Kingdom, USA=United States of America  *Crude rates calculated from presented data  ^1^ St Sauver JL, Grossardt BR, Yawn BP, Melton LJ 3rd, Pankratz JJ, Brue SM, Rocca WA (2012). Data resource profile: the Rochester Epidemiology Project (REP) medical records-linkage system. Int J Epidemiol. Dec; 41(6):1614-24. doi: 10.1093/ije/dys195. | | | | | | | | | | | | |

| **Supplementary Table 3. Further details of studies presenting data on the incidence and/or prevalence of CBS.** | | | | | | | | | | | | |
| --- | --- | --- | --- | --- | --- | --- | --- | --- | --- | --- | --- | --- |
| **Author, date** | **Location** | **Methods to identify possible cases** | **Methods to identify included cases** | **Diagnostic Criteria for CBS** | **Years of data collection** | **Population** | **Cases** | **Incidence of CBS Per 100,000 (95% CI)** | | **Prevalence of CBS Per 100,000 (95% CI)** | | **Subgroups reported a** |
|  |  |  |  |  |  |  |  | **Crude** | **Age adjusted** | **Crude** | **Age adjusted** |  |
| 1. Harvey, 2003 | London, UK | Multiple source  referrals from  health  professionals  Hospital  information  systems and case  registers in four  hospitals | Review of case  records by  psychiatrist | Not  specified | 2003 | 240,766 | 2 |  |  | 0.83  (0.10-3.00)* |  | Participants  <64 |
| 2. Tan, 2004 | Singapore | Door-to-door  survey | Examination by  study physician of  residents testing  positive on  screening tests  (Phase 2) with  subsequent  confirmation at a  movement  disorders clinic | Kumar et al | 2001-2003 | 14,906 | 1 |  |  | 6.67  (0.17-37.14)* |  | Participants  >50 only |
| 3. Winter, 2010 | Moscow,  Russia | Referrals  -primary care  centres  -hospital  personnel  Medical records  -University  Movement  Disorders centre  -primary care  centres  -hospitals | Examination by  study neurologist  with experience in  movement  disorders | Lang 1994 | 2006-2008 | 1,237,900 | 1 | 0.03  (0.01-0.18) | 0.02  (0.01-0.12) |  |  |  |
| 4. Tartari, 2010 | Argentina | Medical records  -surveillance of  electronic medical  records within a  HMO  -pharmacy system  for patients  prescribed  antiparkinsonian drugs | Medical record | Not  specified | Not stated | 148,301 | Not stated |  |  | 2.43  (0-5.98) |  |  |
|  |  |  | review by  movement  disorders  specialists |  |  |  |  |  |  |  |  |  |
| 5. Osaki, 2011 | Japan | Referrals  -Long-term care  insurance system  -public health  office  -medical  insitutions | Examination by a  specialised  neurologist | Kumar 1998 | 2007 | 66,425 | 6 |  |  | 9 (2-16)  Male: 16  (2-29)  Female: 3  (-3-9) | 6 (0-12)  Male 11 (1-22)  Female 2 (3-6) | Male/Female  Age-adjusted  ≥65  Male ≥65  Female ≥65  0-39  40-49  50-59  60-69  70-79  80-89  ≥90  Male 0-39  Male 40-49  Male 50-59  Male 60-69  Male 70-79  Male 80-89  Male ≥90  Female 0-39  Female 40-  49  Female 50-  59  Female 60-  69  Female 70-  79  Female 80-89 |
| 6. Savica, 2013 | USA | Medical record linkage system (Rochester Epidemiology Project) | Review of medical records by a movement disorders specialist | Maraganore et al. | 1991-2005 | Not stated (1,852,762 person-years) | 4 | 0.2 |  |  |  |  |
| 7. Withall, 2014 | Eastern | Structured | Review of  medical records | Not  specified | 2008 | 129,070 | 1 |  |  | 0.77  (0.02-4.32)* |  | Participants |
|  | Sydney, | questionnaire |  |  |  |  |  |  |  |  |  | <65 only |
|  | Australia | distributed to |  |  |  |  |  |  |  |  |  |  |
|  |  | health |  |  |  |  |  |  |  |  |  |  |
|  |  | professionals  Search of hospital records |  |  |  |  |  |  |  |  |  |  |
|  |  |  |  |  |  |  |  |  |  |  |  |  |
|  |  |  |  |  |  |  |  |  |  |  |  |  |
| 8. Caslake, 2014 | Scotland | Referrals  -GPs  -neurologists  -medicine for the  elderly physicians  -old-age psychiatrists  -general physicians Medical records  -screening of referral letters  -GP databases  -Hospital discharge data Questionnaire- based population  screening | Examination by study neurologist  or neurologist in  training | NR | 2002-2006 | 317,357 (1,176,552  person-  years) | 2 | 0.17  (0.02-0.61)* |  |  |  | Male/Female  0-39  40-49  50-59  60-69  70-79  80-89  90+  Male 0-39  Male 40-49  Male 50-59  Male 60-69  Male 70-79  Male 80-89  Male 90+  Female 0-39  Female 40-49  Female 50-59  Female 60-69  Female 70-79  Female 80-89  Female 90+ |
| 9. Khedr, 2015 | Egypt | Door-to-door screening with a questionnaire of a random sample of households | Examination by neurologist | DSM IV | 2011-2013 | 8,027 | 2 |  |  | 25 (0-59) |  |  |
| 10. Coyle-  Gilchrist, 2016 | UK | Referrals from  -Regional specialist clinics  -Clinical research networks  -Self-referral  -Local and national charities Search of clinic databases | Examination by neurologist Structured assessments of speech, language, and cognition MRI of brain | Armstrong 2013 | 2013-2014 | 1,692,345 | 48 | 0.06  (0.01-0.61)* |  | 2.84  (2.09-3.77)* |  | 0-44  45-49  50-54  55-59  60-64  65-69  70-74  75-79  80-84  85-89  90+ |
| 11. Fleury, 2018 | Switzerland | Medical records  -Hospitals  -Private Neurologists  -Nursing homes | Clinical notes and imaging data reviewed by movement disorders specialist | Armstrong 2013 | 2003-2012 | 470,512 | 14 | 0.8 (0.4-1.3)  Male: 0.6  (0.2-1.3)  Female:  0.6 (0.2-1.4) | 1.4 (0.3-2.4)  Male: 1.5  (0.0-3.2)  Female:  1.1  (0.0-2.3) | 3 (1.6-5.0)  Male: 2.6  (1.0-5.7)  Female:  3.3  (1.4-6.5) | 3.2 (1.5-4.8)  Male: 3.0  (0.6-5.4)  Female:  3.0  (0.9-5.1) | Male/Female 0-39  40-49  50-59  60-69  70-79  80-89  90+  Male 0-39  Male 40-49  Male 50-59  Male 60-69  Male 70-79  Male 80-89  Male 90+  Female 0-39  Female 40-  49  Female 50-  59  Female 60-  69  Female 70-  79  Female 80-  89  Female 90+ |
| 12. Stang, 2020 | USA | Medical record linkage system (Rochester Epidemiology  Project) | Case notes reviewed by a movement disorders  specialist | Armstrong 2013 | 1991-2005 | Not stated  Details of REP reported by St Sauver et al:  502,820 | 3 | 0.4 |  |  |  | Male/Female 1991-1995  1996-2000  2001-2005 |
| Abbreviations:  CBS=Corticobasal Syndrome, DSM-IV=Diagnostic and Statistical Manual IV, FTLD=frontotemporal lobar degeneration, H-ICDA=Hospital adaptation of the International Classification of Diseases, ICD-9= International Classification of Disease, Ninth Revision, ICD-10=International Classification of Diseases, Revision 10, NINDS-SPSP=National Institute of Neurological Disorders and Stroke and the Society for PSP, NJ=New Jersey, NNIPPS=The Natural History and Neuroprotection in Parkinson Plus Syndromes, NR=Not reported PSP=Progressive Supranuclear Palsy, UK=United Kingdom, USA=United States of America  *Crude rates calculated from presented data.  ^1^ St Sauver JL, Grossardt BR, Yawn BP, Melton LJ 3rd, Pankratz JJ, Brue SM, Rocca WA (2012). Data resource profile: the Rochester Epidemiology Project (REP) medical records-linkage system. Int J Epidemiol. Dec; 41(6):1614-24. doi: 10.1093/ije/dys195. | | | | | | | | | | | | |

| **Supplementary table 4. Characteristics of included studies assessed by the Joanna Briggs Institute Inventory.** | | | | | | | | | | | |
| --- | --- | --- | --- | --- | --- | --- | --- | --- | --- | --- | --- |
| **No** | **Paper author** | **Year** | **1. Was the sample frame appropriate to address the target population?** | **2. Were the study participants sampled in an appropriate way?** | **3.Was the sample size adequate?** | **4. Were the study subjects and setting described in detail?** | **5. Was the data analysis conducted with sufficient coverage of the identified sample?** | **6. Were valid methods used for the identification of the condition?** | **7. Was the condition measured in a standard, reliable way for all participants?** | **8. Was there appropriate statistical analysis?** | **9. Was the response rate adequate, and if not, was the low response rate managed appropriately?** |
| 1 | Radhnakrisnan | 1988 | Yes | Yes | No | Yes | Yes | Yes | Yes | Yes | Not applicable |
| 2 | Golbe | 1988 | Yes | Yes | Yes | Yes | Yes | Yes | Yes | No | Not applicable |
| 3 | De Rijk | 1995 | Yes | Yes | No | Yes | Yes | Yes | Yes | Yes | Not applicable |
| 4 | Bower | 1997 | Yes | Yes | No | Yes | Yes | Yes | Yes | No | Not applicable |
| 5 | Wermuth | 1997 | Yes | Yes | No | Yes | Yes | Yes | Yes | Yes | Not applicable |
| 6 | Chio | 1998 | Yes | Yes | No | Yes | Yes | Yes | Yes | Yes | Not applicable |
| 7 | Schrag | 1999 | Yes | Yes | No | Yes | Yes | Yes | Yes | Yes | Not applicable |
| 8(a) | Nath | 2001 | Yes | Yes | Yes | No | Yes | Yes | Yes | Yes | Not applicable |
| 8(b) | Nath | 2001 | Yes | Yes | Yes | Yes | Yes | Yes | Yes | Yes | Not applicable |
| 8(c) | Nath | 2001 | Yes | Yes | No | Yes | Yes | Yes | Yes | Yes | Not applicable |
| 9 | Yamada | 2001 | Yes | Yes | No | Yes | Yes | Yes | Unclear | Yes | Yes |
| 10 | Harvey | 2003 | Yes | Yes | Yes | Yes | Yes | Yes | Unclear | Yes | Not applicable |
| 11 | Zhang | 2003 | Yes | Yes | No | Yes | Yes | Yes | Yes | Yes | Yes |
| 12 | Bergareche | 2004 | Yes | Yes | No | Yes | Yes | Yes | Yes | Yes | Yes |
| 13 | Kawashima | 2004 | Yes | Yes | No | Yes | Yes | Yes | Yes | Yes | Not applicable |
| 14 | Tan | 2004 | Yes | Yes | Yes | Yes | Yes | Yes | Yes | Yes | Not applicable |
| 15 | Wermuth | 2008 | Yes | Yes | No | Yes | Yes | Yes | Yes | No | Not applicable |
| 16 | Linder | 2010 | Yes | Yes | No | Yes | Yes | Yes | Yes | Yes | Not applicable |
| 17 | Tatari* | 2010 | Yes | Yes | No | No | Unclear | Unclear | Unclear | Yes | Not applicable |
| 18 | Winter | 2011 | Yes | Yes | Yes | Yes | Yes | Yes | Yes | Yes | Not applicable |
| 19 | Osaki | 2011 | Yes | Yes | No | Yes | Yes | Yes | Yes | Yes | Not applicable |
| 20 | Nakashita* | 2011 | Yes | Yes | No | No | Unclear | Unclear | Unclear | No | Not applicable |
| 21 | Savica | 2013 | Yes | Yes | No | Yes | Yes | Yes | Yes | No | Not applicable |
| 22 | Caslake | 2014 | Yes | Yes | No | Yes | Yes | Yes | Yes | Yes | Not applicable |
| 23 | Withall | 2014 | Yes | Yes | Yes | Yes | Yes | Unclear | Unclear | Yes | Yes |
| 24 | Khedr | 2015 | Yes | Yes | No | Yes | Yes | Yes | Yes | Yes | Not applicable |
| 25 | Coyle-Gilchrist | 2016 | Yes | Yes | Yes | Yes | Yes | Yes | Yes | Yes | Not applicable |
| 26 | Takigawa | 2016 | Yes | Yes | No | Yes | Yes | Yes | Yes | Yes | Not applicable |
| 27 | Fleury | 2018 | Yes | Yes | Yes | Yes | Yes | Yes | Yes | Yes | Not applicable |
| 28 | Calo-Perxas | 2019 | Yes | Yes | Yes | Yes | Yes | Yes | Yes | Yes | Not applicable |
| 29 | Logroscino | 2019 | Yes | Yes | Yes | Yes | Yes | Yes | Yes | Yes | Not applicable |
| 30 | Stang | 2020 | Yes | Yes | No | Yes | Yes | Yes | Yes | Yes | Not applicable |
| 31 | Viscidi* | 2020 | Yes | Yes | Yes | No | Unclear | No | Unclear | No | Not applicable |
| 32 | Viscidi | 2021 | Yes | Yes | Yes | Yes | Yes | No | Yes | No | Not applicable |
| ***Conference abstract only** | | | | | | | | | | | |

**Supplementary Table 5: PRISMA Checklist 2009**

| **Section/topic** | **#** | **Checklist item** | **Reported on page #** |
| --- | --- | --- | --- |
| **TITLE** | | |  |
| Title | 1 | Identify the report as a systematic review, meta-analysis, or both. | Page 1 |
| **ABSTRACT** | | |  |
| Structured summary | 2 | Provide a structured summary including, as applicable: background; objectives; data sources; study eligibility criteria, participants, and interventions; study appraisal and synthesis methods; results; limitations; conclusions and implications of key findings; systematic review registration number. | Page 3 |
| **INTRODUCTION** | | |  |
| Rationale | 3 | Describe the rationale for the review in the context of what is already known. | Page 5 |
| Objectives | 4 | Provide an explicit statement of questions being addressed with reference to participants, interventions, comparisons, outcomes, and study design (PICOS). | Page 6, Supplementary Table 1 |
| **METHODS** | | |  |
| Protocol and registration | 5 | Indicate if a review protocol exists, if and where it can be accessed (e.g., Web address), and, if available, provide registration information including registration number. | Page 6 |
| Eligibility criteria | 6 | Specify study characteristics (e.g., PICOS, length of follow-up) and report characteristics (e.g., years considered, language, publication status) used as criteria for eligibility, giving rationale. | Page 6-7 |
| Information sources | 7 | Describe all information sources (e.g., databases with dates of coverage, contact with study authors to identify additional studies) in the search and date last searched. | Page 7 |
| Search | 8 | Present full electronic search strategy for at least one database, including any limits used, such that it could be repeated. | Table 1 |
| Study selection | 9 | State the process for selecting studies (i.e., screening, eligibility, included in systematic review, and, if applicable, included in the meta-analysis). | Page 6-7 |
| Data collection process | 10 | Describe method of data extraction from reports (e.g., piloted forms, independently, in duplicate) and any processes for obtaining and confirming data from investigators. | Page 7 |
| Data items | 11 | List and define all variables for which data were sought (e.g., PICOS, funding sources) and any assumptions and simplifications made. | Page 7 |
| Risk of bias in individual studies | 12 | Describe methods used for assessing risk of bias of individual studies (including specification of whether this was done at the study or outcome level), and how this information is to be used in any data synthesis. | Page 7 Supplementary Table 4 |
| Summary measures | 13 | State the principal summary measures (e.g., risk ratio, difference in means). | Page 7-8 |
| Synthesis of results | 14 | Describe the methods of handling data and combining results of studies, if done, including measures of consistency (e.g., I^2^) for each meta-analysis. | Page 8 |

| **Section/topic** | **#** | **Checklist item** | **Reported on page #** |
| --- | --- | --- | --- |
| Risk of bias across studies | 15 | Specify any assessment of risk of bias that may affect the cumulative evidence (e.g., publication bias, selective reporting within studies). | Page 7 |
| Additional analyses | 16 | Describe methods of additional analyses (e.g., sensitivity or subgroup analyses, meta-regression), if done, indicating which were pre-specified. | Page 8 |
| **RESULTS** | | |  |
| Study selection | 17 | Give numbers of studies screened, assessed for eligibility, and included in the review, with reasons for exclusions at each stage, ideally with a flow diagram. | Page 9 and Figure 1 |
| Study characteristics | 18 | For each study, present characteristics for which data were extracted (e.g., study size, PICOS, follow-up period) and provide the citations. | Table 2, Supplementary Table 2,3, and 6. |
| Risk of bias within studies | 19 | Present data on risk of bias of each study and, if available, any outcome level assessment (see item 12). | Supplementary Table 4 |
| Results of individual studies | 20 | For all outcomes considered (benefits or harms), present, for each study: (a) simple summary data for each intervention group (b) effect estimates and confidence intervals, ideally with a forest plot. | Figure 2, 4, and 5 |
| Synthesis of results | 21 | Present results of each meta-analysis done, including confidence intervals and measures of consistency. | Figure 4 |
| Risk of bias across studies | 22 | Present results of any assessment of risk of bias across studies (see Item 15). | Supplementary Table 4 |
| Additional analysis | 23 | Give results of additional analyses, if done (e.g., sensitivity or subgroup analyses, meta-regression [see Item 16]). | Figure 5 |
| **DISCUSSION** | | |  |
| Summary of evidence | 24 | Summarize the main findings including the strength of evidence for each main outcome; consider their relevance to key groups (e.g., healthcare providers, users, and policy makers). | Page 12-15 |
| Limitations | 25 | Discuss limitations at study and outcome level (e.g., risk of bias), and at review-level (e.g., incomplete retrieval of identified research, reporting bias). | Page 15 |
| Conclusions | 26 | Provide a general interpretation of the results in the context of other evidence, and implications for future research. | Page 16 |
| **FUNDING** | | |  |
| Funding | 27 | Describe sources of funding for the systematic review and other support (e.g., supply of data); role of funders for the systematic review. | Page 2 |

From: Moher D, Liberati A, Tetzlaff J, Altman DG, The PRISMA Group (2009). Preferred Reporting Items for Systematic Reviews and Meta-Analyses: The PRISMA Statement. PLoS Med 6(7): e1000097. doi:10.1371/journal.pmed1000097 For more information, visit: [www.prisma-statement.org](http://www.prisma-statement.org).

| **Supplementary Table 6: Table of all included papers and abstracts.** | |
| --- | --- |
| 1 | Radhakrishnan K, Thacker AK, Maloo JC, Gerryo SE, Mousa ME (1988) Descriptive epidemiology of some rare neurological diseases in Benghazi, Libya. Neuroepidemiology 7, 159-164. doi:10.1159/000110150 |
| 2 | Golbe LI, Davis PH, Schoenberg BS, Duvoisin RC (1988) Prevalence and natural history of progressive supranuclear palsy. Neurology. 38, 1031-1034. doi:10.1212/wnl.38.7.1031 |
| 3 | de Rijk MC, Breteler MM, Graveland GA, Ott A, Grobbee DE, van der Meche FG, Hofman A (1995) Prevalence of Parkinson's disease in the elderly: the Rotterdam Study. Neurology. 45, 2143-2146. doi:10.1212/wnl.45.12.2143 |
| 4 | Bower JH, Maraganore DM, McDonnell SK, Rocca WA (1997) Incidence of progressive supranuclear palsy and multiple system atrophy in Olmsted County, Minnesota, 1976 to 1990. Neurology. 49, 1284-1288. doi:10.1212/wnl.49.5.1284 |
| 5 | Wermuth L, Joensen P, Bünger N, Jeune B (1997) High prevalence of Parkinson's disease in the Faroe Islands. Neurology 49, 426-432. doi:10.1212/wnl.49.2.426 |
| 6 | Chio A, Magnani C, Schiffer D (1998) Prevalence of Parkinson's disease in Northwestern Italy: comparison of tracer methodology and clinical ascertainment of cases. Mov Disord. 13, 400-405. doi:10.1002/mds.870130305 |
| 7 | Schrag A, Ben-Shlomo Y, Quinn NP (1999) Prevalence of progressive supranuclear palsy and multiple system atrophy: a cross-sectional study. Lancet. 354, 1771-1775. doi:10.1016/s0140-6736(99)04137-9 |
| 8 | Nath U, Ben-Shlomo Y, Thomson RG, Morris HR, Wood NW, Lees AJ, Burn DJ (2001) The prevalence of progressive supranuclear palsy (Steele-Richardson-Olszewski syndrome) in the UK. Brain. 124, 1438-1449. doi:10.1093/brain/124.7.1438 |
| 9 | Yamada T, Hattori H, Miura A, Tanabe M, Yamori Y (2001) Prevalence of Alzheimer's disease, vascular dementia and dementia with Lewy bodies in a Japanese population. Psychiatry Clin Neurosci. 55, 21-25. doi:10.1046/j.1440-1819.2001.00779.x |
| 10 | Harvey RJ, Skelton-Robinson M, Rossor MN (2003) The prevalence and causes of dementia in people under the age of 65 years. J Neurol, Neurosurg, and Psychiatry. 74, 1206-1209. doi:10.1136/jnnp.74.9.1206 |
| 11 | Zhang ZX, Anderson DW, Huang JB, Li H, Hong X, Wei J, Yang EL, Maraganore DM (2003) Prevalence of Parkinson's disease and related disorders in the elderly population of greater Beijing, China. Mov Disord. 18, 764-772. doi:10.1002/mds.10445 |
| 12 | Bergareche A, De La Puente E, Lopez de Munain A, Sarasqueta C, de Arce A, Poza JJ, Marti-Masso JF (2004) Prevalence of Parkinson's disease and other types of Parkinsonism. A door-to-door survey in Bidasoa, Spain. J Neurol. 251, 340-345. doi:10.1007/s00415-004-0333-3 |
| 13 | Kawashima M, Miyake M, Kusumi M, Adachi Y, Nakashima K (2004) Prevalence of progressive supranuclear palsy in Yonago, Japan. Mov Disord. 19, 1239-1240. doi:10.1002/mds.20149 |
| 14 | Tan LC, Venketasubramanian N, Hong CY, Sahadevan S, Chin JJ, Krishnamoorthy ES, Tan AK, Saw SM (2004) Prevalence of Parkinson disease in Singapore: Chinese vs Malays vs Indians. Neurology. 62, 1999-2004. doi:10.1212/01.wnl.0000128090.79756.10 |
| 15 | Wermuth L, Bech S, Petersen MS, Joensen P, Weihe P, Grandjean P (2008) Prevalence and incidence of Parkinson's disease in The Faroe Islands. Acta Neurol Scand. 118, 126-131. doi:10.1111/j.1600-0404.2007.00991.x |
| 16 | Tartari JP SC, Bauso DJ, Giunta D, Rojas JI, Cristiano E. (2010) Prevalence of parkinsonism in a health maintenance organization from Buenos Aires City. Mov Disord. 25, S264. https://doi.org/10.1002/mds.23162 |
| 17 | Linder J, Stenlund H, Forsgren L (2010) Incidence of Parkinson's disease and parkinsonism in northern Sweden: a population-based study. Mov Disord. 25, 341-348. doi:10.1002/mds.22987 |
| 18 | Winter Y, Bezdolnyy Y, Katunina E, Avakjan G, Reese JP, Klotsche J, Oertel WH, Dodel R, Gusev E (2010) Incidence of Parkinson's disease and atypical parkinsonism: Russian population-based study. Mov Disord 25, 349-356. doi:10.1002/mds.22966 |
| 19 | Osaki Y, Morita Y, Kuwahara T, Miyano I, Doi Y (2011) Prevalence of Parkinson's disease and atypical parkinsonian syndromes in a rural Japanese district. Acta Neurol Scand. 124, 182-187. doi:10.1111/j.1600-0404.2010.01442.x |
| 20 | Nakashita S WIK, Uemura Y, Yamawaki M, Tanaka K, Nakashima K. (2011) Parkinsonism in a community dwelling elderly population sample in Japan. Eur J Neurol. 18, 545. https://doi.org/10.1111/ene.14306 |
| 21 | Savica R, Grossardt BR, Bower JH, Ahlskog JE, Rocca WA (2013) Incidence and pathology of synucleinopathies and tauopathies related to parkinsonism. JAMA Neurol. 70, 859-866. doi:10.1001/jamaneurol.2013.114 |
| 22 | Caslake R, Taylor K, Scott N, Harris C, Gordon J, Wilde K, Murray A, Counsell C (2014) Age-, and gender-specific incidence of vascular parkinsonism, progressive supranuclear palsy, and parkinsonian-type multiple system atrophy in North East Scotland: the PINE study. Parkinsonism Relat Disord. 20, 834-839. doi:10.1016/j.parkreldis.2014.04.013 |
| 23 | Withall A, Draper B, Seeher K, Brodaty H (2014) The prevalence and causes of younger onset dementia in Eastern Sydney, Australia. Int Psychogeriatr. 26, 1955-1965. doi:10.1017/S1041610214001835 |
| 24 | Khedr EM, Fawi G, Abbas MA, Mohammed TA, El-Fetoh NA, Attar GA, Zaki AF (2015) Prevalence of Parkinsonism and Parkinson's disease in Qena governorate/Egypt: a cross-sectional community-based survey. Neurol Res 37, 607-618. doi:10.1179/1743132815Y.0000000020 |
| 25 | Coyle-Gilchrist IT, Dick KM, Patterson K, Vazquez Rodriquez P, Wehmann E, Wilcox A, Lansdall CJ, Dawson KE, Wiggins J, Mead S, Brayne C, Rowe JB (2016) Prevalence, characteristics, and survival of frontotemporal lobar degeneration syndromes. Neurology. 86, 1736-1743. doi:10.1212/WNL.0000000000002638 |
| 26 | Takigawa H, Kitayama M, Wada-Isoe K, Kowa H, Nakashima K (2016) Prevalence of progressive supranuclear palsy in Yonago: change throughout a decade. Brain Behav 6, e00557. doi:10.1002/brb3.557 |
| 27 | Fleury V, Brindel P, Nicastro N, Burkhard PR (2018) Descriptive epidemiology of parkinsonism in the Canton of Geneva, Switzerland. Parkinsonism Relat Disord. 54, 30-39. doi:10.1016/j.parkreldis.2018.03.030 |
| 28 | Calvo-Perxas L, Belchi O, Turon-Estrada A, Van Eendenburg C, Linares M, Vinas M, Manzano A, Turro-Garriga O, Vilalta-Franch J, Garre-Olmo J, Registry of Dementia of Girona Study G (2019) Incidence and characteristics of uncommon dementia subtypes: Results from 10 years of clinical surveillance by the Registry of Dementia of Girona. Alzheimers Dement. 15, 917-926. doi:10.1016/j.jalz.2019.03.017 |
| 29 | Logroscino G, Piccininni M, Binetti G, Zecca C, Turrone R, Capozzo R, Tortelli R, Battista P, Bagoj E, Barone R, Fostinelli S, Benussi L, Ghidoni R, Padovani A, Cappa SF, Alberici A, Borroni B (2019) Incidence of frontotemporal lobar degeneration in Italy: The Salento-Brescia Registry study. Neurology. 92, e2355-e2363. doi:10.1212/WNL.0000000000007498 |
| 30 | Stang CD, Turcano P, Mielke MM, Josephs KA, Bower JH, Ahlskog JE, Boeve BF, Martin PR, Upadhyaya SG, Savica R (2020) Incidence and Trends of Progressive Supranuclear Palsy and Corticobasal Syndrome: A Population-Based Study. J Parkinsons Dis. 10, 179-184. doi:10.3233/JPD-191744 |
| 31 | Viscidi E, Morris H, Harrington, A, Inuzaka Y, Li L, Eaton S, Orlovic M. (2020) The epidemiology of progressive supranuclear palsy in the United Kingdom: Evidence from the Clinical Practice Research Datalink GP Online Database (CPRD GOLD). Eur J Neurol. 27:1-102. https://doi.org/10.1111/ene.14306 |
| 32 | Viscidi E, Litvan I, Dam T, Juneja M, Li L, Krzywy H, Eaton S, Hall S, Kupferman J, Hoglinger GU (2021) Clinical Features of Patients With Progressive Supranuclear Palsy in an US Insurance Claims Database. Front Neurol. 12, 571800. doi:10.3389/fneur.2021.571800 |
